# Reductive Functionalization of Amides in Synthesis and for Modification of Bioactive Compounds

**DOI:** 10.3389/fchem.2021.655849

**Published:** 2021-04-26

**Authors:** Paweł J. Czerwiński, Bartłomiej Furman

**Affiliations:** Institute of Organic Chemistry, Polish Academy of Sciences, Warsaw, Poland

**Keywords:** amide reduction, functionalization, bioactive compounds, natural products, late-stage functionalization, bioisosteres

## Abstract

In this review, applications of the amide reductive functionalization methodology for the synthesis and modification of bioactive compounds are covered. A brief summary of the different protocols is presented in the introduction, followed by the synthetic application of these in late-stage functionalization, leading to known pharmaceuticals or to their derivatives, including bioisosteres, with potential higher activity as the main axis of the article. The synthetic approach to natural products based on amide reduction is also discussed; however, this is given in a condensed form focusing on recent or as yet unexplored applications due to a number of recently published excellent reviews covering this topic. The aim of this review is to illustrate the potential of reductive functionalization of amides as an elegant and useful tool in the synthesis of bioactive compounds and inspire further work in this field.

## Introduction

The addition of nucleophiles to amides to obtain substituted amines represents a major challenge, and only scattered applications for particular substrates have appeared. Initial improvements were based on the activation of amides by the introduction of particular substituents, such as *N*-methoxy amides (Weinreb amides) or electron-withdrawing groups able to increase the carbon nucleophilicity (Pace et al., 2014) and enhance the rate of nucleophilic addition in comparison with C–N bond cleavage, which prevents daunting overaddition problem (Li and Szostak, 2020). Although these strategies facilitate the introduction of nucleophiles, chemoselectivity issues arise when additional electrophilic moieties (e.g., carbonyls) are present, thus decreasing the versatility of these methods.

**Figure 1:**
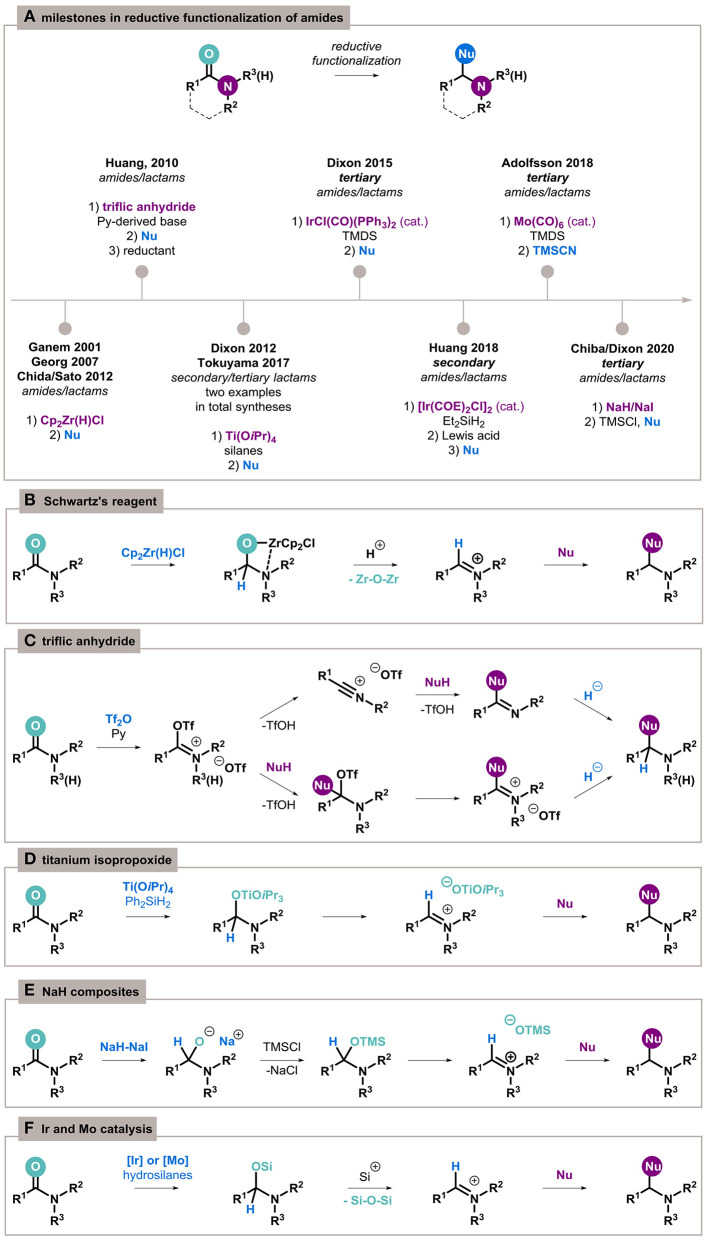
Reductive functionalization of amides overview. **(A)** Key moments in the development of the process. **(B–F)** Mechanistic insights of particular methods.

On the other hand, monoreduction of amides to imines is also challenging but is of interest as an alternative method for the preparation of important compounds and as a potential synthetic pathway for the transformation of inert carbonyl functions. In recent years, important advancements toward chemoselective partial reduction and subsequent functionalization of amides have been made, including the latest report of inorganic reducing system (Figure 1A) (Czerwiński and Furman, 2020).

Relevant to this theme is the seminal example by Ganem (Schedler et al., 1993), extended later by Georg (mechanistic studies) (Spletstoser et al., 2007), Chida and Sato (acyclic amides) (Oda et al., 2012), and Furman (lactams) (Szcześniak et al., 2014) about the conversion of amides to imines by a stoichiometric amount of Schwartz's reagent, Cp_2_Zr(H)Cl. The capture of four-membered zirconacycle intermediates with acids generate highly electrophilic iminium species, which are able to undergo chemoselective addition by a broad range of nucleophiles opening access to substituted amines (Figure 1B).

Amides can also be activated in an electrophilic manner to C–N cleavage (Li et al., 2020) or carbonyl bond functionalization highlighted in this review. Charette laid the foundations for the chemoselective activation of amides with triflic anhydride (Tf_2_O) in the presence of a base (Charette and Grenon, 2001), which was applied for nucleophilic functionalization by Huang (Xiao et al., 2010a). The reaction proceeds differently, depending on the amide structure; however, *O*-triflyliminium triflate intermediates are a commonality (Figure 1C). The addition of the nucleophile results in the formation of a functionalized imine, which can then be reduced by various hydride donors to the final amine.

Two other notable stoichiometric methods include activation by titanium isopropoxide and the transition-metal–free reduction by inorganic NaH/NaI composites. The first, established by Buchwald (Bower et al., 1996), is based on Ti(O*i*Pr)_4_ and dihydrosilanes (Figure 1D). Only two particular examples of its application for functionalization have been reported so far, as key steps in the synthesis of histrionicotoxin by Tokuyama (Sato et al., 2017) and manzamine A by Dixon (Jakubec et al., 2012). Very recently, surprisingly, sodium hydride joined this group because of the specific enhancement of its reducing activity by the formation of a composite with other inorganic components (Figure 1E) (Hong et al., 2016). This methodology can be applied to the exhaustive reduction of amides (Ong et al., 2019), as well as their reductive functionalization (Ong et al., 2020).

Catalytic reduction of amides has also been achieved using iridium catalysts such as [Ir(COE)_2_Cl]_2_ with diethylsilane as a reductant for secondary amides (Cheng and Brookhart, 2012) and IrCl(CO)(PPh_3_)_2_ (Vaska's complex) with tetramethyldisiloxane (TMDS) for tertiary amides (Motoyama et al., 2009). The process proceeds through an *N,O*-silylacetal generated by the iridium-enabled hydrosilylation of the carbonyl bond. Treating the reaction mixture with acid can transform acetal to imine or iminium ions, and subsequent addition of a nucleophilic agent allows for the preparation of highly functionalized amines (Figure 1F). Recently, a highly chemoselective Mo(CO)_6_/TMDS catalytic system proceeding in the same manner was proposed by Adolfsson (Tinnis et al., 2016; Trillo et al., 2018).

## Natural Products Synthesis

Natural products have long been known to be an excellent source of bioactive compounds as illustrated by the fact that 28% of the drugs approved to date have a natural origin or are a derivative of such (Laraia et al., 2018). Its synthesis is always a challenging task for synthetic chemists. Every transformation requires the smart utilization of functional groups in order to reduce protecting group use and reduce process complexity, which is worth its weight in gold. The reductive functionalization of amides belongs to this type of processes and could be a highly useful shortcut, especially in the complex syntheses of natural products. An excellent review covering nucleophilic addition to amides, including the reduction/addition approach, was published some time ago by Chida and Sato, who presented selected recent advances in this field (Sato et al., 2018). In addition, very recently, Dixon and coworkers summarized the application of iridium-catalyzed reactions of tertiary amides for the synthesis of complex amine building blocks and natural products (Matheau-Raven et al., 2020). In contrast, this section is dedicated to all types of amides and only to the application of reductive functionalization of amides in natural products synthesis and is focused on examples omitted from or published after the appearance of the mentioned reviews.

**Figure 2:**
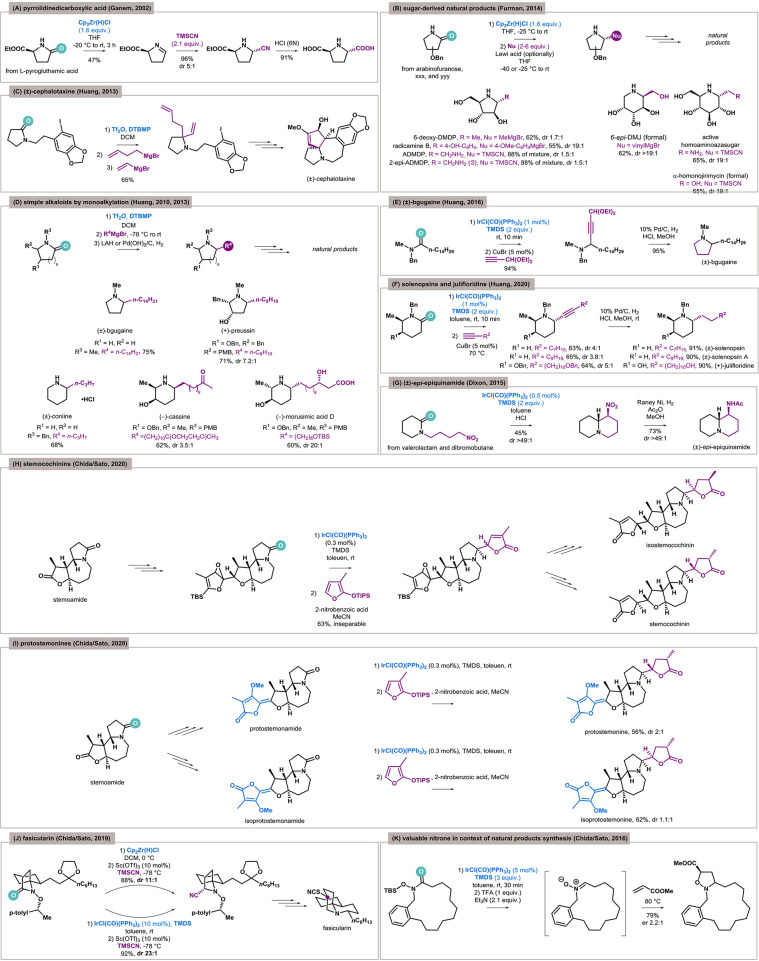
Reductive functionalization of amides as a key step in natural products synthesis. **(A)** Synthesis of a pyrrolidine-based alkaloid by zirconocene hydrochloride. **(B)** The first application of Schwartz's reagent in a reduction of sugar-derived lactams. **(C)** Synthesis of (±)-cephalotaxine via triflic anhydride–mediated bisalkylation. **(D)** Application of the triflic anhydride based monoalkylation protocol in the synthesis of selected alkaloids. **(E)** Iridium-catalyzed approach to bgugaine by an addition/ring closure sequence. **(F)** Approach to iridium-catalyzed piperidine-based alkaloids. **(G)** Synthesis of (±)-*epi*-epiquinamide via cyclization of *N*-linked lactams by Vaska's complex. **(H)** An iridium-catalyzed key step of stemocochinins total synthesis. **(I)** A crucial step in protostemonine total synthesis enabled by Vaska's complex. **(J)** A total synthesis of fasicularin with the iridium-catalyzed key transformation. **(K)** Nitrone synthesis from amides allowed by Vaska's complex.

The first seminal report in this field was the synthesis of kainic acid by Ganem in 2001 (Xia and Ganem, 2001). This was the first application of reductive functionalization of an amide as a key step in natural product synthesis and has been extensively described in many reviews. However, it is noteworthy that in the following year the methodology was also applied to the synthesis of (2*S*,5*S*)-pyrrolidine-2,5-dicarboxylic acid (Figure 2A) isolated from the red alga *Schizymenia dubyi* (Xia and Ganem, 2002).

In 2014, Furman and coworkers presented the Schwartz's reagent–mediated reduction of sugar-derived lactams for the first time (Szcześniak et al., 2014). This opened a simple approach to functionalized polyhydroxylated cyclic amines, which are important scaffolds of many natural products and iminosugars—carbohydrate mimetics (Figure 2B). The authors applied the developed methodology in the synthesis of 6-deoxy-DMDP and radicamine B (inhibitors of various glycoside hydrolases) (Merino et al., 2008) by functionalization of a benzylated arabinofuranose–derived lactam. The first, isolated from the seeds of the African legume *Angylocalyx pynaertii*, was obtained in 2014 by addition of appropriate Grignard reagent with good yield (62%) but poor diastereoselectivity (dr 1.7:1). A similar pathway was implemented for the second, isolated from the Chinese herb *Lobelia chinensis*, and good yield (55%) and excellent diastereoselectivity (dr > 19:1) were obtained.

The same protocol was utilized for vinylation (62%, dr 20:1) in the formal synthesis of 6-epi-deoxymannojiromycin (6-epi-DMJ), another α-glucosidase inhibitor from plants of the mulberry family (Szcześniak et al., 2014). The utilization of the cyanide anion and arabinofuranose-derived lactam in the same manner gave access to amino-DMDP (Szcześniak, 2014). The stereoselectivity was unsatisfactory (88%, dr 1.5:1); however, both diastereomers were described as useful in the treatment of Fabry disease (Cheng et al., 2016). In turn, the same reaction with glucose-derived lactam furnishes a functionalized piperidine (65%, dr > 19:1) (Szcześniak, 2014), which can be reduced to aminopiperidine with strong glucosidase inhibition activity (Wong et al., 1995). Moreover, a possible transformation to CH_2_OH gives access to α-homonojirmycin, another iminosugar isolated from a wide range of plants.

In 2010, Huang applied electrophilic activation of amides by Tf_2_O for the reductive vicinal alkylation of lactams (Xiao et al., 2010a). In the same year, the methodology was improved and selective monoaddition became possible (Xiao et al., 2010b). Both protocols allow for easy access to simple pyrrolidine and piperidine alkaloids. Based on this, Huang and coworkers synthesized (±)-cephalotaxine by double alkylation (Figure 2C) (Xiao et al., 2013a). On the other hand, the monoalkylation protocol allowed the synthesis of several structurally similar alkaloids (Figure 2D): (±)-bgugaine (a hepatotoxin and an antibacterial and antimycotic agent isolated from the tubers of *Arisarum vulgare*), (±)-coniine (poison isolated from *Conium maculatum*), (±)-cassine (antibacterial agent isolated from leaves of *Senna racemosa*), (+)-preussin (antifungal agent and a growth inhibitor of fission yeast and human cancer cells isolated from *Preussia* fungi), and morusimic acid D (α-glucosidase inhibitor isolated from *Morus alba*) (Xiao et al., 2010b, 2013b).

In 2016, Huang and coworkers published a protocol for the reductive alkynylation of amides enabled by dual Ir/Cu catalysis (Huang et al., 2016). The reduction of tertiary amides by a IrCl(CO)(PPh_3_)_2_/TMDS system and subsequent alkynylation catalyzed by CuBr gave a propargylic amine containing a ketal functional group. Its hydrogenation under acidic conditions resulted in simultaneous debenzylation of the nitrogen atom, reduction of alkyne, deprotection of carbonyl form, and reductive amination with cyclization to the pyrrolidine core (Figure 2E). This sequence is complementary to a previous Huang's approach to (±)-bgugaine (Xiao et al., 2010b).

In 2020, Huang and coworkers applied this alkynylation methodology for the functionalization of lactams. Practically, the same tactic as previously described was applied, and alkylation/reduction was used as an alternative to the mono/bis alkylation approach for the synthesis of noncomplex alkaloids—(±)-solenopsin, (±)-solenopsin A, and (+)-julifloridine (Figure 2F) (Ou et al., 2020). Solenopsins are components of the venom of the fire ant *Solenopsis invicta*. The structurally appropriate *N*-benzyl tertiary amides were reduced and alkynylated. In this way, *N*-α,α′-branched piperidines were obtained with good trans-selectivity (3.8–4:1). Full hydrogenation of the triple bond gave finally the target products. On the other hand, the synthesis of (+)-julifloridine was based on (*R*)-*N*-benzyl-3-benzyloxy glutarimide, which was initially diastereoselectively methylated to an α-branched lactam. The reductive alkynylation by *O*-benzylated alkylacetylene in a trans-selective manner (5:1) and subsequent deprotection of hydroxyl functions gave (+)-julifloridine.

The first iridium-catalyzed functionalization, the reductive nitro-Mannich cyclization of *N*-linked lactams, was reported in 2015 by Dixon (Gregory et al., 2015). The sequence of reduction of lactam to enamine, its reprotonation to iminium ion under acidic conditions, and addition/cyclization of the generated nitronate ion gave a series of condensed biheterocycles of various sizes. One of these was exploited in the synthesis of (±)-*epi*-epiquinamide (Figure 2G) (Gregory et al., 2015), an alkaloid with as yet unidentified activity isolated from the skin extracts of an Ecuadorian poisonous frog *Epipedobates tricolor* (Sangsuwan et al., 2017).

In 2020, Chida and Sato (Soda et al., 2020) applied the reductive functionalization of tertiary lactams to the synthesis of a family of stemoamide-type alkaloids first isolated from *Stemonaceae* plants with medicinal and antiparasitic activity (Alibés and Figueredo, 2009). The strategy was based on the chemoselective addition of lactones to lactams starting from stemoamide (Figure 2H). The process was challenging because of the presence of a highly electrophilic and more reactive lactone in the scaffold. The iridium-catalyzed chemoselective functionalization of lactam by 2-siloxyfuran in the presence of an epoxy function unfortunately furnished an inseparable mixture of diastereoisomers with combined 63% yield, which was separated after the next step to pure hemiacetals (47% yield for major, 32% for the minor product), which suggests the diastereoselectivity of reductive functionalization on level 1.5:1. Further transformations gave natural products with pyrrolidine and pyrrolidine core (after oxidation). The strategy for related protostemonines synthesis was similar (Figure 2I), and the diastereoselectivities were also unsatisfactory (2:1 for protostemonine, 1.1:1 for isoprotostemonine).

In 2019, Chida and Sato presented an elegant synthetic pathway to fasicularin, a cytotoxic alkaloid isolated from *Nephteis fasicularis* (Weinreb, 2006), based on the reductive functionalization of a chiral *N*-alkoxy lactam as a key step (Figure 2J) (Yamamoto et al., 2019). The synthesis started with preinstallation of a (*S*)-1-(4-methylphenyl)ethoxy moiety on the nitrogen atom of the lactam to perform a highly diastereoselective Strecker reaction. Two methods of lactam activation were examined—Schwartz's reagent and IrCl(CO)(PPh_3_)_2_/TMDS system. The first gave good stereoselectivity (88%, dr 11:1); however, the iridium catalyst turned out to be even better and furnished the desired nitrile with excellent results (92%, dr 23:1). The cyano group incorporated in this way was reduced to an alcohol and then transformed to thiocyanate, which allows the construction of fasicularin by ingenious ring expansion through opening of the intermediated aziridine.

Under the “reductive functionalization” term, the construction of a new C–C/C–Het bond in place of the carbonyl is expected by the synthetic community, rather than an exhaustive reduction by overaddition of hydride anions (Khalimon et al., 2019). This process can, however, also afford highly desired products, such as nitrones (Murahashi and Imada, 2019). It is worth highlighting the excellent protocol by Chida and Sato, which is based on nitrone formation from *N*-hydroxyamides (Katahara et al., 2016). This class of amides, activated by an iridium complex, undergoes hydrosilylation of the carbonyl group and desilylation/elimination of the hydroxyl group to form a nitrone. The developed methodology was applied to the formation of macrocyclic nitrones (Figure 2K), key intermediates for the synthesis of biologically active natural products such as manzamine alkaloids (Brandi et al., 2017) and the β-lactam core (Stecko et al., 2014). Moreover, in this case, an *N*-silyloxyamide was used as a substrate, and a 15-membered cyclic nitrone was captured by cycloaddition with methyl acrylate to form an enantiomeric isoxazolidine.

## Late-Stage Functionalization

The definition of late-stage functionalization was not clear until recently, and it could be narrowed down only to C–H functionalization by chemists out of the field. Of course, C–H functionalization can be an excellent example of late-stage functionalization, provided it meets specified requirements. Last year, Ritter made an attempt to define late-stage functionalization based on the analysis of representative examples (Börgel and Ritter, 2020). Two main factors should characterize the process: (1) chemoselectivity and (2) absence of directing or activating group previously incorporated to the molecule for this purpose. These specifications result in the definition of late-stage functionalization being “a desired chemoselective transformation on a complex molecule to provide at least one analog in sufficient quantity and purity for a given purpose without the necessity for the installation of a functional group that exclusively serves the purpose to enable said transformation” (Börgel and Ritter, 2020). Of course, the “complexity” is still hard to determine and often remains intuitive. In principle, late-stage functionalization should provide easy access to modification of significant molecules to improve their potential value and is dedicated mainly, but not only to medicinal or materials chemistry. In this context, the reductive functionalization of amides perfectly fits into Ritter's definition (Börgel and Ritter, 2020) and due to this has started to play an important role in drug development over recent years. This transformation is applied both in the synthesis of bioactive compounds as well as a tool for their modification, and in this context, it will be presented in this section.

### Late-Stage Functionalization to Bioactive Compounds

**Figure 3:**
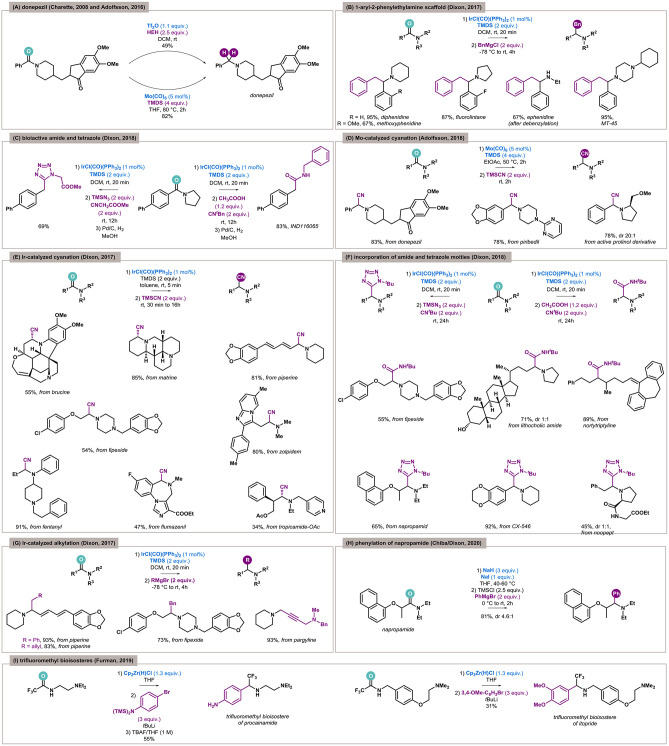
Reductive functionalization of amides in context of late-stage functionalization and bioisosteres. **(A)** Comparison of triflic anhydride–mediated and molybdenum-catalyzed late-stage exhaustive reduction of amide to donepezil. **(B)** Iridium-catalyzed benzylation of amides to bioactive aryl-phenethylamine scaffold. **(C)** Reductive Ugi-type modifications for bioactive molecules by late-stage functionalization of amide via iridium catalysis. **(D)** Molybdenum-catalyzed late-stage cyanation of selected drugs. **(E)** Iridium-catalyzed late-stage cyanation of alkaloids and drugs. **(F)** Late-stage transformation of bioactive amides to α-amino amides and tetrazoles. **(G)** Late-stage alkylation of active compounds. **(H)** Late-stage phenylation of herbicide by Chiba and Dixon's protocol based on NaH/NaI composite. **(I)** The first example of synthesis of bioisosteres by reductive functionalization of amides.

Although exhaustive reduction by overaddition of hydride anions is much easier than functionalization by the construction of a new C–C/C–Het bond, the unique chemoselectivity of the developed methodologies means this type of transformation could be highly desired. The most explored example of this is the synthesis of donepezil, an acetylcholinesterase inhibitor used in the treatment of Alzheimer disease. In 2008, Charette used electrophilic activation by Tf_2_O for this purpose, and the reduction of benzamide to benzylamine in the presence of an active ketone was achieved with moderate yield (49%) (Barbe and Charette, 2008). A Mo(CO)_6_/TMDS system turned out to be more effective and proceeded with very good yield (82%, Figure 3A) (Tinnis et al., 2016).

In 2017, Dixon presented coupling of different types of Grignard reagents with tertiary amides catalyzed by IrCl(CO)(PPh_3_)_2_ (Xie and Dixon, 2017). The developed methodology was applied to the synthesis of drugs containing the 1-aryl-2-phenylethylamine scaffold—antineurodegenerative agents diphenidine, methoxphenidine, and fluorolintane, as well as anesthetic ephenidine and analgesic MT-45 (Figure 3B). In all cases, the benzyl moieties came from Grignard reagents, and the tertiary amide was reduced. Ephenidine is a secondary amine, and its synthesis included the removal of an *N*-benzyl group in the final step.

In 2018, Dixon again exploited tertiary amides in Ugi-type reactions between *in situ*–formed iminium species, isocyanide, and (thio)acetic acid or trimethylsilyl azide (Xie and Dixon, 2018). The process furnishes α-amino (thio)amides and tetrazoles as products and can be utilized in the synthesis of pharmaceutical agents or for their functionalization. In the case of bioactive molecules synthesis, the pyrrolidine-derived biarylamide was used as a substrate to obtain α-aminoamide and α-aminotetrazole. In the next step, deamination in the presence of palladium on carbon under an atmosphere of hydrogen yielded inhibitors of cell viability and anandamide cellular uptake, respectively (Figure 3C).

### Late-Stage Functionalization of Bioactive Compounds

In 2018, Adolfsson showed that the Mo(CO)_6_-catalyzed reduction was compatible with amide carbonyl replacement by a cyanide function (Trillo et al., 2018). Amides were utilized as precursors for the synthesis of functionalized drugs containing the benzylamine moiety. Again, donepezil was the compound of interest, as well as the antiparkinsonian agent piribedil and prolinol derivatives possessing significant biological activity (Figure 3D). In this way, late-stage cyanated analogs of valuable drugs were efficiently obtained (78–83%). In 2017, Dixon and coworkers also reported the iridium-catalyzed reductive Strecker reaction (Arriba et al., 2017), and a late-stage cyanation of a few drugs and alkaloids containing an amide moiety was presented (Figure 3E). From the alkaloid family, an analgesic but also toxic brucine found in *Strychnos nux-vomica* trees, antitumural matrine isolated from plants belonging to the *Sophora* genus and responsible for the spicy of *Piper nigrum* (black pepper), and a piperine with various activities were exploited. A vast group of drugs—psychoactive fipexide and zolpidem, acylated antimuscarinic tropicamide, γ-aminobutyric acid receptor antagonist flumazenil, and anesthetic fentanyl—were also successfully cyanated.

Again, Dixon applied the aforementioned iridium-catalyzed transformation of tertiary amides to α-aminoamides and α-aminotetrazoles in Ugi-type reactions (Xie and Dixon, 2018). The methodology was used for the synthesis of medicinally relevant inhibitors and also for the late-stage functionalization of various active compounds. The α-aminoamide moiety was incorporated into the structure of the psychoactive agent fipexide, amide of the anticarcinogen lithocholic acid, and a derivative of the antidepressant nortriptyline. In turn, tetrazole derivatives were made from herbicidal napropamide, antineurodegenerative candidate CX-546, and nootropic noopept (Figure 3F).

As already mentioned, in 2017 Dixon showed the iridium-catalyzed reductive functionalization of tertiary amides by Grignard reagents (Xie and Dixon, 2017). Besides the synthesis of several drugs, late-stage functionalization of bioactive compounds was also presented—benzylated and homoallylated piperine, benzylated fipexide, and a pargyline derivative containing a methylene piperidine motif were obtained with good to excellent yields (54–93%, Figure 3G).

Very recently, Chiba and Dixon again utilized Grignard reagents as nucleophiles. However, this time the unique sodium hydride–sodium iodide composite was used as the reducing system (Ong et al., 2020). The developed methodology was applied for the late-stage functionalization of napropamide (Figure 3H), a soil-applied herbicide for pre-emergence control of weeds in a range of crops. The phenylated napropamide was obtained with very good yield and in a diastereoselective manner (81%, dr 4.6:1).

## Bioisosteres Synthesis

The reductive functionalization of amides opens a simple route to the installation of moieties, which could mimic carbonyl function and be amide bioisosteres. The thoughtful choice of replacement gives a high probability of more efficient candidates or significant improvement of marketed drugs (Kumari et al., 2020).

For amides, a number of classes are known, including tetrazole, fluoroalkene, or trifluoroethylamine. Nevertheless, the reductive functionalization of amides has only been employed to afford trifluoroethylamine-based bioisosteres to date (Czerwiński and Furman, 2019). The unique electronic properties and small size of the fluorine atom make it a versatile bioisostere for a lone pair of electrons, the hydrogen atom, and the methyl group, as well as the carbonyl, hydroxyl, and nitrile functionalities (Meanwell, 2018). In the context of physiochemical properties, fluorine incorporation can have an impact on conformation, pKa, intrinsic potency, membrane permeability, metabolic pathways, and pharmacokinetic properties (Gillis et al., 2015).

The successful application of trifluoroethylamine unit as amide and peptide bioisostere lies in its unique basicity properties. This group serves to neutralize the nucleophilicity of the alkylamine (Sani et al., 2007). According to this, the nitrogen atom has low Lewis basicity and is a relatively poor hydrogen-bond acceptor. On the other hand, the proton attached to the nitrogen is a good hydrogen bond donor because of the strong electron-withdrawing effect induced by the trifluoromethyl group. These factors make the trifluoroethylamine moiety similar to the amide/peptide bond. However, not only similarities, but also differences are important. The CF_3_ group is a much weaker hydrogen-bond acceptor in comparison to the carbonyl oxygen; therefore, the trifluoroethylamine function can play the role of amide bond bioisostere only when the carbonyl group of the original molecule is not part of a significant hydrogen-bonding system with the receptor. The second deviation is in the shape of compounds. It is clear that amide moiety is planar, and trifluoroethylamine is tetrahedral. The consequences of this fact can be surprising—CF_3_ as a relatively bulky group (comparable to isopropyl) (Belot et al., 2017) can affect the active conformation and force subtle position changes of other substituents, in addition to shielding the molecule from metabolism, which results in a positively prolonged time of drug release.

In 2019, Furman and Czerwiński published a protocol for reductive functionalization of secondary fluoroacetamides (Czerwiński and Furman, 2019). As an illustration of the synthetic potential of the developed methodology, the synthesis of trifluoroethylamine-based bioisosteres of two marketed drugs containing amide moieties was presented. The key step involved Schwartz's reagent, which turned out to be selective for reactive fluorinated amides and bioisosteres of antiarrhythmic procainamide and prokinetic itopride was successfully obtained (Figure 3I).

## Conclusions and Outlook

Methods of selective activation of amides and lactams discovered in the last two decades have enabled access to novel reactivity pathways and have opened up intriguing perspectives in the synthesis of bioactive compounds. Demonstrations of possibilities of reductive functionalization of amides have been presented in this review in order to both highlight their utility for the synthesis of target scaffolds and their promise in increasing the biological activity of existing and new drugs. The ubiquity and stability of the amide group have allowed the developed methodologies to be directly applicable for the late-stage functionalization of a wide range of biologically active molecules. Unfortunately, despite their obvious advantages, the discussed methodology has not yet found industrial applications, but the change of this state seems to be highly probable and will be breakthrough for the field.

## Author Contributions

PC collected the related references and prepared the manuscript. BF directed the preparation of this manuscript. Both authors critically reviewed the text and figures prior to submission.

## Conflict of Interest

The authors declare that the research was conducted in the absence of any commercial or financial relationships that could be construed as a potential conflict of interest.

## References

[B1] Alibés R. Figueredo M. (2009). Strategies for the synthesis of stemona alkaloids. Eur. J. Org. Chem. 2009, 2421–2435. 10.1002/ejoc.200900037

[B2] Arriba Á. L. F. D. Lenci E. Sonawane M. Formery O. Dixon D. J. (2017). Iridium-catalyzed reductive strecker reaction for late-stage amide and lactam cyanation. Angew. Chem. Int. Ed. 56, 3655–3659. 10.1002/anie.201612367 28233919

[B3] Barbe G. Charette A. B. (2008). Highly chemoselective metal-free reduction of tertiary amides. J. Am. Chem. Soc. 130, 18–19. 10.1021/ja077463q 18076177

[B4] Belot V. Farran D. Jean M. Albalat M. Vanthuyne N. Roussel C. (2017). Steric scale of common substituents from rotational barriers of N-(o-substituted aryl)thiazoline-2-thione atropisomers. J. Org. Chem. 82, 10188–10200. 10.1021/acs.joc.7b01698 28901766

[B5] Börgel J. Ritter T. (2020). Late-stage functionalization. Chem 6, 1877–1887. 10.1016/j.chempr.2020.07.007

[B6] Bower S. Kreutzer K. A. Buchwald S. L. (1996). A mild general procedure for the one-pot conversion of amides to aldehydes. Angew. Chem. Int. Ed. 35, 1515–1516. 10.1002/anie.19961515123978737

[B7] Brandi A. Cardona F. Cicchi S. Cordero F. M. Goti A. (2017). [3 + 2] Dipolar cycloadditions of cyclic nitrones with alkenes. Org. React. 94, 1–321. 10.1002/0471264180.or094.01

[B8] Charette A. B. Grenon M. (2001). Spectroscopic studies of the electrophilic activation of amides with triflic anhydride and pyridine. Can. J. Chem. 79, 1694–1703. 10.1139/v01-150

[B9] Cheng C. Brookhart M. (2012). Iridium-catalyzed reduction of secondary amides to secondary amines and imines by diethylsilane. J. Am. Chem. Soc. 134, 11304–11307. 10.1021/ja304547s 22770123

[B10] Cheng W.-C. Wang J.-H. Li H.-Y. Lu S.-J. Hu J.-M. Yun W.-Y.. (2016). Bioevaluation of sixteen ADMDP stereoisomers toward alpha-galactosidase A: development of a new pharmacological chaperone for the treatment of fabry disease and potential enhancement of enzyme replacement therapy efficiency. Eur. J. Med. Chem. 123, 14–20. 10.1016/j.ejmech.2016.07.025 27474919

[B11] Czerwiński P. J. Furman B. (2019). Overcoming inaccessibility of fluorinated imines – synthesis of functionalized amines from readily available fluoroacetamides. Chem. Commun. 55, 9436–9439. 10.1039/C9CC04111G 31304490

[B12] Czerwiński P. J. Furman B. (2020). A long-sought reactivity of a sodium hydride. Trends Chem. 2, 782–784. 10.1016/j.trechm.2020.07.001

[B13] Gillis E. P. Eastman K. J. Hill M. D. Donnelly D. J. Meanwell N. A. (2015). Applications of fluorine in medicinal chemistry. J. Med. Chem. 58, 8315–8359. 10.1021/acs.jmedchem.5b00258 26200936

[B14] Gregory A. W. Chambers A. Hawkins A. Jakubec P. Dixon D. J. (2015). Iridium-catalyzed reductive nitro-mannich cyclization. Chem. Eur. J. 21, 111–114. 10.1002/chem.201405256 25399919 PMC4730865

[B15] Hong Z. Ong D. Y. Muduli S. K. Too P. C. Chan G. H. Tnay Y. L.. (2016). Understanding the origins of nucleophilic hydride reactivity of a sodium hydride–iodide composite. Chem. Eur. J. 22, 7108–7114. 10.1002/chem.201600340 27038135

[B16] Huang P.-Q. Ou W. Han F. (2016). Chemoselective reductive alkynylation of tertiary amides by Ir and Cu(i) bis-metal sequential catalysis. Chem. Commun. 52, 11967–11970. 10.1039/C6CC05318A 27722243

[B17] Jakubec P. Hawkins A. Felzmann W. Dixon D. J. (2012). Total synthesis of manzamine A and related alkaloids. J. Am. Chem. Soc. 134, 17482–17485. 10.1021/ja308826x 23039372

[B18] Katahara S. Kobayashi S. Fujita K. Matsumoto T. Sato T. Chida N. (2016). An iridium-catalyzed reductive approach to nitrones from N-hydroxyamides. J. Am. Chem. Soc. 138, 5246–5249. 10.1021/jacs.6b02324 27071479

[B19] Khalimon A. Y. Gudun K. A. Hayrapetyan D. (2019). Base metal catalysts for deoxygenative reduction of amides to amines. Catalysts 9:490. 10.3390/catal9060490

[B20] Kumari S. Carmona A. V. Tiwari A. K. Trippier P. C. (2020). Amide bond bioisosteres: strategies, synthesis, and successes. J. Med. Chem. 63, 12290–12358. 10.1021/acs.jmedchem.0c00530 32686940 PMC7666045

[B21] Laraia L. Robke L. Waldmann H. (2018). Bioactive compound collections: from design to target identification. Chem 4, 705–730. 10.1016/j.chempr.2018.01.012

[B22] Li G. Ma S. Szostak M. (2020). Amide bond activation: the power of resonance. Trends Chem. 2, 914–928. 10.1016/j.trechm.2020.08.001

[B23] Li G. Szostak M. (2020). Kinetically controlled, highly chemoselective acylation of functionalized grignard reagents with amides by N–C cleavage. Chem. Eur. J. 26, 611–615. 10.1002/chem.201904678 31696589

[B24] Matheau-Raven D. Gabriel P. Leitch J. A. Almehmadi Y. A. Yamazaki K. Dixon D. J. (2020). Catalytic reductive functionalization of tertiary amides using vaska's complex: synthesis of complex tertiary amine building blocks and natural products. ACS Catal. 10, 8880–8897. 10.1021/acscatal.0c02377

[B25] Meanwell N. A. (2018). Fluorine and fluorinated motifs in the design and application of bioisosteres for drug design. J. Med. Chem. 61, 5822–5880. 10.1021/acs.jmedchem.7b01788 29400967

[B26] Merino P. Delso I. Tejero T. Cardona F. Marradi M. Faggi E.. (2008). Nucleophilic additions to cyclic nitrones en route to iminocyclitols – total syntheses of DMDP, 6-deoxy-DMDP, DAB-1, CYB-3, nectrisine, and radicamine B. Eur. J. Org. Chem. 2008, 2929–2947. 10.1002/ejoc.200800098

[B27] Motoyama Y. Aoki M. Takaoka N. Aoto R. Nagashima H. (2009). Highly efficient synthesis of aldenamines from carboxamides by iridium-catalyzed silane-reduction/dehydration under mild conditions. Chem. Commun. 13, 1574–1576. 10.1039/b821317h 19277394

[B28] Murahashi S.-I. Imada Y. (2019). Synthesis and transformations of nitrones for organic synthesis. Chem. Rev. 119, 4684–4716. 10.1021/acs.chemrev.8b00476 30875202

[B29] Oda Y. Sato T. Chida N. (2012). Direct chemoselective allylation of inert amide carbonyls. Org. Lett. 14, 950–953. 10.1021/ol3000316 22260368

[B30] Ong D. Y. Fan D. Dixon D. J. Chiba S. (2020). Transition-metal-free reductive functionalization of tertiary carboxamides and lactams for α-branched amine synthesis. Angew. Chem. Int. Ed. 59, 11903–11907. 10.1002/anie.202004272 32329555

[B31] Ong D. Y. Yen Z. Yoshii A. Revillo Imbernon J. Takita R. Chiba S. (2019). Controlled reduction of carboxamides to alcohols or amines by zinc hydrides. Angew. Chem. Int. Ed. 58, 4992–4997. 10.1002/anie.201900233 30761712

[B32] Ou W. Lu G.-S. An D. Han F. Huang P.-Q. (2020). Two-step catalytic transformation of N-benzyllactams to alkaloids (±)-solenopsin, (±)-solenopsin A, and (+)-julifloridine. Eur. J. Org. Chem. 2020, 52–56. 10.1002/ejoc.201901752

[B33] Pace V. Holzer W. Olofsson B. (2014). Increasing the reactivity of amides towards organometallic reagents: an overview. Adv. Synth. Catal. 356, 3697–3736. 10.1002/adsc.201400630

[B34] Sangsuwan W. Kongkathip B. Chuawong P. Kongkathip N. (2017). Total synthesis of (+)-epiquinamide and (–)-epiepiquinamide from d-mannose. Tetrahedron 73, 7274–7281. 10.1016/j.tet.2017.11.016

[B35] Sani M. Volonterio A. Zanda M. (2007). The trifluoroethylamine function as peptide bond replacement. ChemMedChem 2, 1693–1700. 10.1002/cmdc.200700156 17823898

[B36] Sato M. Azuma H. Daigaku A. Sato S. Takasu K. Okano K.. (2017). Total synthesis of (–)-histrionicotoxin through a stereoselective radical translocation–cyclization reaction. Angew. Chem. Int. Ed. 56, 1087–1091. 10.1002/anie.201609941 27990730

[B37] Sato T. Yoritate M. Tajima H. Chida N. (2018). Total synthesis of complex alkaloids by nucleophilic addition to amides. Org. Biomol. Chem. 16, 3864–3875. 10.1039/C8OB00733K 29701231

[B38] Schedler D. J. A. Godfrey A. G. Ganem B. (1993). Reductive deoxygenation by Cp_2_ZrHCl: selective formation of imines via zirconation/hydrozirconation of amides. Tetrahedron Lett. 34, 5035–5038. 10.1016/S0040-4039(00)60669-X

[B39] Soda Y. Sugiyama Y. Yoritate M. Tajima H. Shibuya K. Ogihara C.. (2020). Unified total synthesis of pentacyclic stemoamide-type alkaloids. Org. Lett. 22, 7502–7507. 10.1021/acs.orglett.0c02697 32960064

[B40] Spletstoser J. T. White J. M. Tunoori A. R. Georg G. I. (2007). Mild and selective hydrozirconation of amides to aldehydes using Cp_2_Zr(H)Cl: scope and mechanistic insight. J. Am. Chem. Soc. 129, 3408–3419. 10.1021/ja066362+ 17315870 PMC2626624

[B41] Stecko S. Furman B. Chmielewski M. (2014). Kinugasa reaction: an ugly duckling of β-lactam chemistry. Tetrahedron 70, 7817–7844. 10.1016/j.tet.2014.06.024

[B42] Szcześniak P. (2014). Wykorzystanie cyklicznych imin jako bloków budulcowych w syntezie izydyn. [dissertation]. Warsaw: Institute of Organic Chemistry Polish Academy of Sciences.

[B43] Szcześniak P. Stecko S. Staszewska-Krajewska O. Furman B. (2014). Sugar-derived cyclic imines: one-pot synthesis and direct functionalization. Tetrahedron 70, 1880–1888. 10.1016/j.tet.2014.01.039

[B44] Tinnis F. Volkov A. Slagbrand T. Adolfsson H. (2016). Chemoselective reduction of tertiary amides under thermal control: formation of either aldehydes or amines. Angew. Chem. Int. Ed. 55, 4562–4566. 10.1002/anie.201600097 26934055

[B45] Trillo P. Slagbrand T. Adolfsson H. (2018). Straightforward α-amino nitrile synthesis through Mo(CO)6-catalyzed reductive functionalization of carboxamides. Angew. Chem. Int. Ed. 57, 12347–12351. 10.1002/anie.201807735 30084524

[B46] Weinreb S. M. (2006). Studies on total synthesis of the cylindricine/fasicularin/lepadiformine family of tricyclic marine alkaloids. Chem. Rev. 106, 2531–2549. 10.1021/cr050069v 16771458 PMC2529480

[B47] Wong C.-H. Provencher L. Porco J. A. Jung S.-H. Wang Y.-F. Chen L.. (1995). Synthesis and evaluation of homoaza sugars as glycosidase inhibitors. J. Org. Chem. 60, 1492–1501. 10.1021/jo00111a007

[B48] Xia Q. Ganem B. (2001). Asymmetric total synthesis of (–)-α-kainic acid using an enantioselective, metal-promoted ene cyclization. Org. Lett. 3, 485–487. 10.1021/ol007009q 11434316

[B49] Xia Q. Ganem B. (2002). An efficient synthesis of substituted prolines by the selective reduction and reductive cyanation of 2-pyrrolidones. Tetrahedron Lett. 43, 1597–1598. 10.1016/S0040-4039(02)00097-7

[B50] Xiao K.-J. Luo J.-M. Xia X.-E. Wang Y. Huang P.-Q. (2013a). General one-pot reductive gem-bis-alkylation of tertiary lactams/amides: rapid construction of 1-azaspirocycles and formal total synthesis of (±)-cephalotaxine. Chem.-Eur. J. 19, 13075–13086. 10.1002/chem.201302096 23956001

[B51] Xiao K.-J. Luo J.-M. Ye K.-Y. Wang Y. Huang P.-Q. (2010a). Direct, one-pot sequential reductive alkylation of lactams/amides with grignard and organolithium reagents through lactam/amide activation. Angew. Chem. Int. Ed. 49, 3037–3040. 10.1002/anie.201000652 20301159

[B52] Xiao K.-J. Wang Y. Huang Y.-H. Wang X.-G. Huang P.-Q. (2013b). A direct and general method for the reductive alkylation of tertiary lactams/amides: application to the step economical synthesis of alkaloid (–)-morusimic acid D. J. Org. Chem. 78, 8305–8311. 10.1021/jo4007656 23909394

[B53] Xiao K.-J. Wang Y. Ye K.-Y. Huang P.-Q. (2010b). Versatile one-pot reductive alkylation of lactams/amides via amide activation: application to the concise syntheses of bioactive alkaloids (±)-bgugaine, (±)-coniine, (+)-preussin, and (–)-cassine. Chem. Eur. J. 16, 12792–12796. 10.1002/chem.201002054 20938943

[B54] Xie L.-G. Dixon D. J. (2017). Tertiary amine synthesis via reductive coupling of amides with Grignard reagents. Chem. Sci. 8, 7492–7497. 10.1039/C7SC03613B 29163902 PMC5676097

[B55] Xie L.-G. Dixon D. J. (2018). Iridium-catalyzed reductive Ugi-type reactions of tertiary amides. Nat. Commun. 9:2841. 10.1038/s41467-018-05192-7 30026608 PMC6053461

[B56] Yamamoto S. Komiya Y. Kobayashi A. Minamikawa R. Oishi T. Sato T.. (2019). Asymmetric total synthesis of fasicularin by chiral N-alkoxyamide strategy. Org. Lett. 21, 1868–1871. 10.1021/acs.orglett.9b00478 30817163

